# Environmental DNA Metabarcoding: A Novel Contrivance for Documenting Terrestrial Biodiversity

**DOI:** 10.3390/biology11091297

**Published:** 2022-08-31

**Authors:** Shahnawaz Hassan, Sabreena, Peter Poczai, Bashir Ah Ganai, Waleed Hassan Almalki, Abdul Gafur, R. Z. Sayyed

**Affiliations:** 1 Department of Environmental Science, University of Kashmir, Srinagar 190006, India; 2 Finnish Museum of Natural History, University of Helsinki, 00014 Helsinki, Finland; 3 Centre of Research for Development, University of Kashmir, Srinagar 190006, India; 4 Department of Pharmacology, College of Pharmacy, Umm Al-Qura University, Makkah 21955, Saudi Arabia; 5 Sinarmas Forestry Corporate Research and Development, Perawang 28772, Indonesia; 6 Department of Microbiology, PSGVP Mandal’s, SI Patil Arts, G B Patel Science and STKV Sangh Commerce College, Shahada 425409, India

**Keywords:** biodiversity conservation, environmental DNA, soil eDNA, community characterization, terrestrial ecosystems

## Abstract

**Simple Summary:**

The innovative concept of environmental DNA has found its foot in aquatic ecosystems but remains an unexplored area of research concerning terrestrial ecosystems. When making management choices, it is important to understand the rate of eDNA degradation, the persistence of DNA in terrestrial habitats, and the variables affecting eDNA detectability for a target species. Therefore an attempt has been made to provide comprehensive information regarding the exertion of eDNA in terrestrial ecosystems from 2012 to 2022. The information provided will assist ecologists, researchers and decision-makers in developing a holistic understanding of environmental DNA and its applicability as a biodiversity monitoring contrivance.

**Abstract:**

The dearth of cardinal data on species presence, dispersion, abundance, and habitat prerequisites, besides the threats impeded by escalating human pressure has enormously affected biodiversity conservation. The innovative concept of eDNA, has been introduced as a way of overcoming many of the difficulties of rigorous conventional investigations, and is hence becoming a prominent and novel method for assessing biodiversity. Recently the demand for eDNA in ecology and conservation has expanded exceedingly, despite the lack of coordinated development in appreciation of its strengths and limitations. Therefore it is pertinent and indispensable to evaluate the extent and significance of eDNA-based investigations in terrestrial habitats and to classify and recognize the critical considerations that need to be accounted before using such an approach. Presented here is a brief review to summarize the prospects and constraints of utilizing eDNA in terrestrial ecosystems, which has not been explored and exploited in greater depth and detail in such ecosystems. Given these obstacles, we focused primarily on compiling the most current research findings from journals accessible in eDNA analysis that discuss terrestrial ecosystems (2012–2022). In the current evaluation, we also review advancements and limitations related to the eDNA technique.

## 1. Introduction

Assessing classical and extant biodiversity is conventionally anticipated by morphological and behavioral data obtained utilizing direct surveys, microscopes, binoculars, traps, and, most recently, bioacoustics [1]. These methods are often biased, intrusive, and/or predisposed by plummeting pool of taxonomic specialists for recognizing specimens [2]. Moreover, traditional surveys are mostly labor demanding and tedious and can be inefficacious at describing the accurate biodiversity in attendance [3]. The emergence of expeditious and moderately affordable DNA sequencing techniques has notably inflated biodiversity exploration and analysis by getting the better of labor-exhaustive long-established assessments and increased the latitude and scope to coherently distinguish biodiversity on a real-time basis utilizing systematized approaches [4].

Among the different techniques for biodiversity assessment, environmental deoxyribonucleic acid (eDNA), the complex mixture of genomic DNA obtained from an environmental sample, is becoming a key component of the ecologists’ and environmental managers’ toolbox, alluring worldwide attention [5,6]. The science of eDNA provides the opportunity to scrutinize the dynamics of species, populations and communities and map their geographical distribution over large scales as well as over long periods. It has the potential to revolutionize conservation science [7]. Environmental DNA uses standard, reproducible and auditable criteria that accurately identify target organisms in different environments [3,8], offering broad taxonomic extensiveness and real-time biodiversity assessment for a multitude of species [9].

The first investigation of environmental DNA based on the microbial diversity from the lake sediments was reported by Ogram et al. (1987) [10]. Within a short duration of time, the concept of environmental DNA witnessed tremendous growth. In the year 1990, an investigation disseminated and analyzed the diversity of 16S rRNA gene in bacterioplankton sampled from the Sargasso Sea using PCR amid cloning [11]. To discern the new pathways, metagenomics was used in uncultured microorganisms where cloning and sequencing of soil eDNA fragments were done [12]. Notable work on DNA metabarcoding published in 2003 described the extraction of megafaunal (mammoth, bison horse), ancient plant, and extinct ratite moa DNA from permafrost [13]. Next-generation sequencing (NGS) development after 2005 rendered the costly and time-consuming cloning phase unnecessary [14]. By 2010, DNA barcoding was stretched out to macroorganisms for diet analysis [15] and then for soil eDNA studies [16]. Over the years, different ways have been applied for eDNA analysis for targeting single species, standard or quantitative PCR for detecting all taxa from a given taxonomic group. PCR based assays are vital such as for bacteria [17], fungi [18], plants [19], eukaryotes [20], fishes [21] and so on.

Although eDNA-centered analysis has rapidly gained momentum in freshwater ecology, its success has been underestimated among terrestrial ecosystems [22]. Several variables affect how easily organisms may be detected [23]. When making management choices, it is important to understand the rate of eDNA degradation, the low end of detection, and the variables affecting eDNA detectability for a target species [24]. Terrestrial eDNA is often used as a metric to quantify population distributions. Understanding the detection and variation of terrestrial eDNA across various species or taxonomic groups will be necessary for making these decisions [25]. The understanding of and possibility of using terrestrial eDNA as a tool in biodiversity conservation are limited by the deposition and degradation of eDNA in both historic and modern terrestrial ecosystems [26].

There have been numerous investigations on terrestrial biodiversity assessment during the last few years [27,28], and it seems a field of infinite possibilities for scientific exploration. However, to achieve the desirable outcomes, it is necessary to design repeatable and reliable biodiversity surveys based on eDNA. It is important to understand the constraints and caveats that affect its implementation [29]. After thoroughly understanding its strengths and limitations, it can be possible to generate correct inferences from the data.

This review paper covers the recent methods for eDNA analysis and the gaps that restrict its inherent value. Additionally, an attempt has been made to analyze the existing literature (2012–2022) to form a baseline for further research involving eDNA in terrestrial ecosystems. 

## 2. Research Methodology

A comprehensive literature search was carried out based on specific keywords using several scientific databases (PubMed, Web of Science (WOS), Scopus). The major search terms include eDNA, monitoring, community, terrestrial ecosystems, metabarcoding, and soil eDNA. The article search was restricted to 2012–2022. These searches were filtered to meet the review’ s objectives after carefully analyzing their subjective abstracts with emphasis on justification. The contents and citations of the chosen literature in peer-reviewed journals further attest to its high quality. As a result, certain journal papers related to the review theme were chosen, and their results were thoroughly discussed.

## 3. Persistence, Detectability and Mobility of Terrestrial eDNA

The fate and behavior of DNA in the medium of terrestrial environments continue to be an inadequately comprehended phenomenon due to the dearth of required interest for eDNA investigations outside the microbial world [30]. Environmental DNA remains detectable in soil for days to years (even decades) when the environmental conditions are not favorable for its degradation [31]. The study by Anderson et al. [32], indicated that amplifiable DNA from camels six years was recovered after the species had left the area by analyzing eDNA from soil samples collected from zoos and farms. The study concluded that deeper soil strata preserved soil eDNA longer, and soil eDNA was a better indicator of above-ground composition of the existing vertebrate community [32]. Replication, frequency and timing of sampling are the essential parameters that should be kept in mind while performing the eDNA investigations. Replication in eDNA-based monitoring can be identified by employing rarefaction or asymptotic richness estimators [33]. Too little or too much replication can result in reduced abilities to detect significant changes in species richness or community composition and wastage of resources [34]. Therefore it is important to use scoping studies to conduct a power analysis to calculate the number of replicates needed to detect a given effect size [34]. In some cases, taking more samples may be recommended but not analyzing them unless needed. One of the greatest advantages of environmental DNA metabarcoding is the reduced frequency and more flexible timing of surveys. However, in terrestrial ecosystems, there are still many unknowns regarding seasonality and eDNA. Therefore further may be required to control variation between different seasons and to provide complete equivalency between different ecosystems.

How well vertebrate eDNA could be detected is highly influenced by factors like host-social organization, behavior and biomass [32]. Yaccoz et al. in another study, monitored crop eDNA in previously cultivated pine fields and found an affirmative relationship between the crop amplicons frequency and the year of crop abandonment.

Though terrestrial ecosystems can be seen as the depository of past biodiversity exploited to reconstruct past ecosystems [35], the modern and historical signals conveyed by the soil can be difficult to disentangle, leading to a higher risk of false positive detection for contemporary species [36,37]. However, one should be mindful that the total soil eDNA usually corresponds to the current material, as proven by the study carried out by Zinger et al. (2009), studying seasonal shifts of communities using soil eDNA.

**Figure 1:**
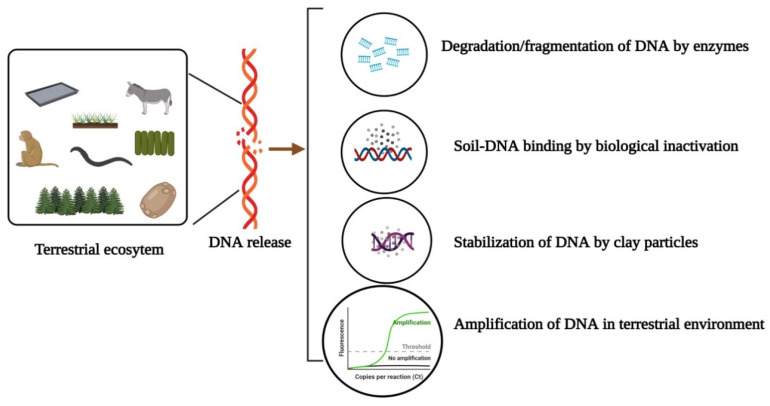
Fate and mobility of DNA in a terrestrial environment.

The spatial diffusion of DNA in a land ecosystem is greatly hindered as it is adsorbed to a substrate such as clay and particles or organic material (Figure 1) [38]. Terrestrial DNA can be obtained using different substrates, and the choice of substrate influence the rate of detection [39]. Multiple factors can help narrow down the most effective substrate for biodiversity surveys. These factors include conditions of the local environment, ecology of the targeted taxa, collection and storage, and time and length of the investigation. The local environment is a notable factor that can alter the availability and suitability of the substrates. For example, snow can be utilized for detecting rare carnivores but only in colder climates [40]. Similarly, scat collection can be suitable for detecting vertebrate diversity in environments with less vegetation cover [41]. Although soil is the prominent substrate employed for the eDNA metabarcoding, local climate can influence its persistence [39,42]. Therefore it is important to validate the methods using pilot studies for a particular ecosystem. The lesser the eDNA in soil, the lesser its signal will be shared between two adjacent communities as the spatial distribution of soil organisms is intrinsically highly structured horizontally and vertically [43]. Horizontal observation has been observed with either traditional methods or DNA metabarcoding [44,45]. In the same way, there is a vertical discrepancy in the community composition of fungi, bacteria and meiofauna between organic and mineral horizons of soil [46,47]. The probability of species detection in a substrate is affected by various factors that influence the rate of DNA deposition and its interaction with that substrate [48]. Larger organisms, for example, will shed more DNA into the environment and can be detected more likely; however, in some reptiles, the keratinized skin may limit the DNA deposition and make their detection difficult [49]. The ecology of target taxa may be considered in evaluating the optimal sample substrate for eDNA monitoring. Hence, applying multiple substrates may be necessary for more elucidated and comprehensive organism monitoring. This has profound implications for the sampling design. In environments where eDNA diffuses poorly, as in soil, terrestrial ecologists should know that sampling effort will always be more intense. However, using composite soil samples can increase the spatial representativeness of soil sampling, hence limiting experimental costs [50]. However, such a design should be chosen if it does not compromise the study’ s success. In addition to scale, the spatial variation in ecosystems can have implications for monitoring as it affects the significance of analyses and should be accounted for in sample design [51]. The utilization of chronosequences, sites of different restoration ages, is common in restoration monitoring as they enable assessment of ecosystem over time [52]. However, the applicability of such design is undermined when there is variation due to spatial autocorrelation. Therefore it is essential to include multiple spatially separated, reference sites to determine the magnitude of the variation that can be attributed either to distance or restoration [39].

From these considerations, we have understood that the soil matrix is a poor indicator of the whole local biodiversity compared to aquatic ecosystems [53]. However, the successful and desired results could be obtained in terrestrial ecosystems using sampler organisms like organisms that feed on plants, arthropods, or even vertebrate skin, carcasses, blood or feces [54,55].

## 4. Exertion of Environmental DNA in Terrestrial Ecosystems 

**Figure 2:**
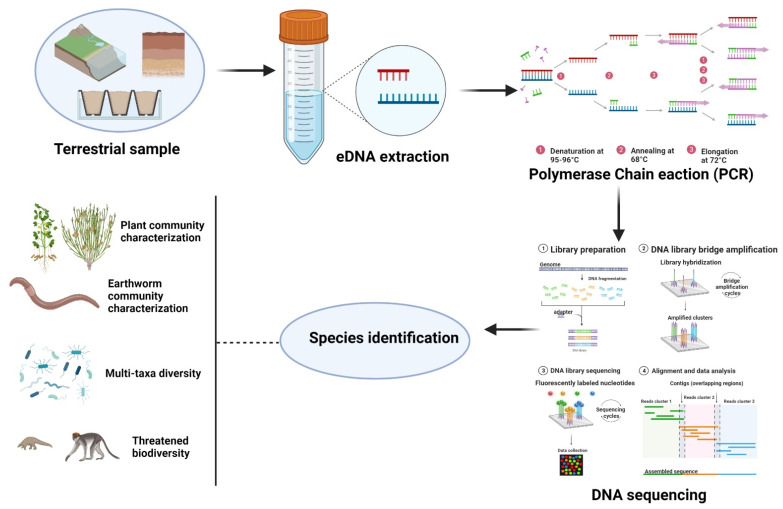
Environmental DNA in terrestrial ecosystems for biodiversity characterization.

There are diverse predominant sources of terrestrial eDNA like soil, saltlicks or any excavated bulk specimen (Figure 2). A terrestrial soil sample can be used to detect indigenous soil species as well as other non-soil-dwelling species. Subsequently, other associations in direct contact with the soil have been detected and studied (Table 1) [56]. These pioneering investigations are well-defined and could not be possible to explore without the introduction of eDNA metabarcoding [57]. Conventional techniques for studying biodiversity help assess and recognize the threats to global ecosystems, with rapidly evolving innovative techniques like eDNA providing novel prospects for all-encompassing biodiversity research besides proving to be a proficient and cost-effective method for evaluating the ecosystem structure and functioning [58,59].

There are probably fewer possibilities for DNA to diffuse in terrestrial systems, making it harder to decide where to sample but less difficult to deal with spatial scale [60]. As a result, DNA is probably concentrated in places where animals spent time or traveled, necessitating a more focused and exact sampling strategy. DNA may become undetectable in water within days to weeks [61], a contrast to possibly years in soils and sediments, since DNA degradation rates are often quicker and less variable in aquatic systems [32,62]. Consequently, it may be difficult to ensure that DNA taken from a terrestrial ecosystem accurately represents species diversity [63]. 

### 4.1. Plant Community Characterization

There is a considerable corpus of knowledge on plant communities gathered by the conventional above-ground botanical inventories that can be constructed with patterns inferred from plant eDNA found in soil [64]. Environmental DNA is a promising tool for identifying vigorous and quiescent seeds, pollen and detritus of species, thereby providing an extensive perspective of plant diversity [65]. This plant community structure can act as a noteworthy aid in outlining the ecological status of the soils [66]. A study carried out by Yacooz et al. [19], indicated that boreal plant communities can be reconstructed by using a short fragment of the P6 loop of the chloroplast trnL (UAA) intron amplified from soil DNA [67]. The results showed high consistency with the data obtained using classical botanical surveys. Yet, for plants, soil DNA is more representative of biomass turnover than actual biomass [68]. This highlights that DNA read frequencies can be difficult to relate to taxon abundances without a proper calibration [69]. This finding is significant as it recognized the limitation of using short DNA barcoding regions for absolute taxonomic resolve [70].

Likewise, there is comparatively a smaller number of investigations carried out on terrestrial eDNA as a contrivance for identifying, examining and/or analyzing plant pathogens. But eDNA techniques can be a potential tool for plant pathogen investigations dealing with identifying and analyzing pathogens [71,72]. For example, if a particular plant is showing symptoms, but no directed examination is possible at that stage, eDNA can come as a rescue for appropriate disease diagnosis [73]. Environmental DNA can also be used for monitoring infectious propagules. It could be an important caveat tool that will allow well-timed and absolute treatment of affected plants before the symptoms become noticeable. In another case, fungal pathogens were recognized in cities and agricultural fields to elucidate the potential for primary warning systems by sampling air [74]. However, one of the biggest challenges of using eDNA methods for identifying plant pathogens is that the genetic resolution of marker genes, in many cases, should be able to distinguish the strains (pathogenic and non-pathogenic). Such challenges have prompted the researchers to use markers that have high resolutions; for example, in *Fusarium*, to increase the resolution, an elongation factor-based marker has been used [75].

### 4.2. Earthworm Community Characterization

Because of their burrowing and casting activities, earthworms are among soil’s most important ecosystem engineers [76]. They play a significant role in nutrient cycling, water retention, and soil fertility [77]. Due to their presence in a substantial proportion of soil animal biomass, the earthworm is sensitive to factors like land use and contamination and hence are considered good indicators of soil health [78].

Earthworm inventories traditionally rely on either passive or physical separation from soil or behavioral methods where earthworms are forced to the surface through physical or chemical stimulus [79]. But these traditional methods are invasive and time-consuming, requiring strong taxonomic skills. The results can be skewed by factors like soil properties, earthworm life stage and species characteristics [80].

The ultimate solution to such queries is to confront the earthworm community studies directly on soil samples using eDNA metabarcoding. Bienert et al. [79], conducted a pioneer study when they designed two metabarcodes regions in the mitochondrial 16sRNA gene specific to earthworms and tested them on French Alps soils detecting endogeic species efficiently. However, as eDNA metabarcoding samples did not contain a leaf litter layer, they missed several epigeic species. After this study, Pansu et al. [81], improved the sampling protocol vertically, including the leaf litter in soil samples they collected and increasing the sample scheme horizontal representativeness covering the entire surface of the studied area. This spatial heterogeneity allowed more detection of species in French Alps soils. This study also proved that earthworm community composition is significantly affected by land use, a pattern that was not brought to light by classical survey methods [81]. In another study, it has been revealed that environmental DNA can be applied to monitor earthworms in agroecosystems, where it was able to detect more species per sample when compared with hand-sorting [82]. Similarly, a nested PCR method in Canada’s boreal forest allowed strong detection of earthworms in archival soil samples stored for up to 30 years [83].

Though there is a tremendous increase in sequencing throughputs, the incompleteness of the reference databases of mitochondrial 16S or RNA gene remains a major impediment to the precise taxonomic identification of earthworms.

### 4.3. Bacterial Community Characterization

Soil microbiologists are probably the most receptive audience for the opportunities offered by the eDNA approaches relying on DNA sequencing to characterize microbial [84] and functional biodiversity [85] for the benefit of the planet and humankind [86].

Brian et al. [87], examined bacterial taxonomic biodiversity at different spatial scales to habitat scale (>10 m) up to global scale. Despite extreme variability, it was found that higher alpha diversity existed in fertilized plots. From the study, it was observed that 20% of the molecular taxonomic units overlap with other EMP samples collected around the world, and the figure reached 40% considerably only in EMP grassland soils, highlighting the existence of a core set of the cosmopolitan bacterial groups [87,88].

Fierer et al. [89], carried out a study to characterize the soil functional diversity and bacterial and archeal taxonomic composition for 16 sites in a wide range of biomass involving shotgun sequencing and 16srDNA sequencing. The results obtained from the study indicated that the desert biome (hot as well as cold) clearly showed apart from other biomes for both metagenome and bacterial community composition, indicating that in a desert environment, bacterial community structure is mainly determined by abiotic conditions instead of microbe-microbe competition [89]. Investigations have revealed that eDNA has the potential to measure microbial communities and forest biodiversity. Therefore leveraging these methods will enhance our ability to detect extant species, describe new species and improve our understanding of ecological and community dynamics in forest ecosystems [50]. In another study, it has been demonstrated that eDNA underpins the great promise that could represent soil microbial eDNA metabarcoding for monitoring restoration progress and success [90].

### 4.4. Multi-Taxa Diversity Surveys

One of the fascinating opportunities offered by eDNA metabarcoding is the possibility of carrying out multi-taxa diversity surveys using the same sampling scheme and eDNA extracts [91]. Soil eDNA metabarcoding can be employed to detect eukaryotic diversities in retort to environmental fluctuations [31].

To analyze how fungal populations in soil and leaf litter responded to a bark beetle-caused tree dieback, Stursova et al. [92] employed metabarcoding. According to their study, the composition of fungal communities altered due to the loss of root-assist fungi and the rise in saprotrophic species, resulting in a drop in the biomass of these communities. [92].

Ramirez et al. [93], investigated biodiversity and biographic patterns from 600 soil cores collected in Central Park, New York City. They studied all three domains of life (bacteria, archaea and eukaryotes) utilizing 16sRNA gene amplification and sequencing for bacterial and archeal diversities and 18srRNA gene for eukaryotic diversity. The results obtained from the study showed that Central Park, an urban and managed ecosystem harbor, had an unscripted level of below-ground biodiversity for all three domains of life, much of which had never been described in public databases [93]. However, these results should be considered with caution as it is difficult to assess how raw data were filtered to discard PCR and sequencing artifacts from the study. These two parameters can greatly inflate biodiversity estimates [94]. Furthermore, it is unclear from the study whether the taxonomic and phylogenetic resolution of the metabarcodes (i.e., 90-bp long for bacteria) was appropriate to allow significant biographical patterns.

Multi-taxa eDNA surveys allow the comprehension of the factors governing soil community assembly and diversity [95]. For example, Zinger et al. [45], examined the fine-grained special distribution of soil bacteria, archaea and eukaryotes in a tropical rainforest plot using eDNA metabarcoding. The study found that soil community composition was highly variable, poorly explained by collected data, and suggested an overall random distribution of soil organisms. The study indicated a differential role of body size on soil community assembly across the tree of life and explains how the integration of diversity census across multiple taxonomic groups can help to test previous hypotheses on complex patterns of biodiversity and community structure [96].

### 4.5. Endangered Species

Only a few studies have been able to trace animals using the eDNA technique from soil [97] in areas where the animals were previously present under controlled conditions like Safari Parks or Zoos or from natural zones where the species are reported [98]. Similarly, an investigation revealed that the endangered New Mexico meadow jumping mouse (*Zapus hudsonius luteus*) is a prime candidate for creating a terrestrial eDNA detection tool because it is restricted to herbaceous riparian zones [99]. More study is necessary to create a reliable survey technique employing this eDNA detection methodology. Our research showed that mammalian eDNA might stay on nest vegetation for a very long time even after the animal has left, underscoring the potential of utilizing eDNA from plants to identify uncommon or threatened terrestrial species. Although it has been shown that eDNA can be a valuable tool for identifying invasive, cryptic, and/or decreasing species, this method is nevertheless constrained by the same limitations that apply to the interpretation of data from conventional survey approaches (e.g., imperfect detection). A quick and efficient way to track distribution and abundance is needed for the wood turtle, a cryptic semi-aquatic species in decline over much of its range [100].

Drammund et al. [101] studied eukaryotic species disparity above and below ground utilizing eDNA from the soil but were unable to identify endangered species [101]. Other investigations used iDNA (invertebrate-derived DNA) from insects like Carrion flies or leeches to monitor terrestrial mammal biodiversity [102]. Schnell et al. [103] succeeded in discovering two species described recently, the Truong Son muntjac (*Muntiacus truongsonensis*) and Annamite stripped rabbit (*Nesolagus timminsi*) [103].

Mammals and potentially endangered species can also be detected using samples from natural saltlicks utilizing eDNA metabarcoding [104]. Using eDNA from the soil to trace mammals [105] is not only environment dependent but also dependent on mammal abundance and size [106]. Hence, knowledge of the ecological behavior of the mammal is essential for the sampling design [99]. We anticipate that eDNA technology will be crucial in delivering quick and widespread insights into the population genetics of endangered and challenging-to-sample species worldwide [49]. 

### 4.6. Bulk Specimens

Traditional ecosystem evaluations based on morphology or barcodes have been employed for terrestrial areas. But as these techniques are expensive, laborious, and less suitable for bulky samples, they allow bulk specimen eDNA metabarcoding to be executed in such ecosystems [107]. Yu et al. [108], provided techniques for classifying bulk arthropod samples using metabarcoding by creating seven arthropod communities and evaluating the richness and composition of the two datasets. They discovered that, although taxonomic information was affected to some extent, eDNA metabarcoding can accurately quantify community differences and diversities of bulk samples [108]. Ji et al. [109] verified bulk arthropod metabarcoding by comparing it to three high-quality reference datasets from Malaysia, China, and the United Kingdom. Metabarcoding produced equivalent statistical models in the same taxon, identified treatments and responses, and connected estimates of species richness for all sites [109]. In another related work, Gibson et al. [110], tested the potential of metabarcoding to characterize diversity by using numerous universal primers on bulk arthropod samples. They discovered that 91 percent of the arthropods could be recognized using metabarcoding, which could also identify microorganisms connected to the arthropods. They also discovered that eDNA metabarcoding was superior to previous approaches and significantly decreased the period and expense of biodiversity research, building a perfect tool for a range of other ecological applications, such as macro and micro-biome interactions [110]. Evaluating host-parasite and community interactions within biodiversity studies, a field of research crucial to biodiversity monitoring but challenging to identify using conventional techniques, may also benefit from the metabarcoding of bulk specimens. Sigut et al. [111] tested the feasibility of metabarcoding for identification using mock samples of insect larvae and parasitoids. They evaluated the completeness of the barcode database by comparing it to a known host-parasitoid database for the research region. In this study, metabarcoding could reliably identify taxa et all taxonomic levels and correctly identify 92.8 percent of all species present in mock samples. Furthermore, they discovered that 39.4% of parasitoid and 90.74% of host taxa could be recognized using the reference database, demonstrating the clear necessity for expanding the parasitoid database. This study’s metabarcoding data indicates more parasitoid diversity than that found in conventional surveys, demonstrating the potential of metabarcoding to discern between the variety of species and to get further accurate identifications via dependable archives [111]. To evaluate the effectiveness of the methodology aimed at vertebrate findings, Rodgers et al. [112], investigated vertebrate-specific metabarcoding of carrion flies in a region with a distinguished mammal diversity. They linked the metabarcoding results to diurnal transect counts and camera trapping. Metabarcoding was able to identify more species than other techniques used concurrently during the same period in each survey (including fly collection), but overall the number of species found was lower when the data from the previous seven years of surveys were taken into account. [112]. The findings of this research indicated that metabarcoding is a useful and competent method for investigating biodiversity, particularly when combined with current monitoring approaches [113]. On the other hand, comprehensive reference databases, improved PCR processes, numerous markers, more extensive sampling efforts, method validation, and accurate sequencing methods are all necessary [114]. Similarly, further research must be done on the terrestrial ecosystem before it can be copiously included in programs for monitoring terrestrial biodiversity [28].

**Table 1:** Representative sample of publications (2012–2022) with major findings based on the application of eDNA in terrestrial ecosystems.

Reference	Ecosystem	Species/Sample	Utility	Major Findings
Ryan et al. (2022) [106]	Terrestrial	Vertebrate species	Suitability of fallen log hollowed sediment as a source of vertebrate eDNA	Environmental DNA (eDNA) monitoring is affected by substrate selection, sampling frequency and size of the animal.
Lyman et al. (2022) [99]	Herbaceous vegetation	Endangered rodent (*Zapus hudsonius luteus*)	Validation of qPCR assay	The feasibility of detecting eDNA from the vegetation is imperative to the life history of New Mexico meadow jumping mice. Environmental DNA can corroborate site occupancy or aid in population density inferences.
Campbell et al. (2022) [91]	Terrestrial	Cryptic insects (*Cochlicella acuta, Sarcophaga villeneuveana*)	Cryptic biological control agent	From a small vegetation sample, the eDNA technique has the potential to detect to infer the presence of cryptic species.
Peterson et al. (2022) [105]	Terrestrial	Invasive terrestrial insect (*Lycorma delicatula*)	Species monitoring	For the detection of lanternfly (*Lycorma delicatula*) insects, deploying roller surface eDNA methods can provide improved guidance for surveillance and monitoring programs.
Lunghi et al. (2022) [57]	Terrestrial	Springtails and insects	Metabarcoding	Environmental DNA from cave soils/sediments acts as a conveyer belt of biodiversity information.
Kirtane et al. (2022) [96]	Forest	*Adelges tsugae*, *Leucotaraxis piniperda*, *L argenticollis*, *L nigrinus*	Forest pest and biological control predators	Environmental DNA as a sensitive biodiversity monitoring tool has greater efficacy for the early detection of *Adelges tsugae* and its biological control predators.
Guerrieri et al. (2021) [31]	Soil	bacteria, fungi and eukaryotes	Effects of soil preservation for biodiversity monitoring	A preserved soil sample can be utilized in metabarcoding research focusing on inaccessible or difficult-to-reach places.
Allen et al. (2021) [35]	Agricultural ecosystem	Invasive pest insect (*Lycorma delicatula)*	Terrestrial eDNA survey	When spotted lanternflies were present in a plot, the likelihood of finding them with eDNA was 84%, more than twice as likely as using visual surveys (36 percent).
Valentin et al. (2021) [53]	Terrestrial	Arthropods	Above-ground terrestrial eDNA	An increase in filter pore size had no discernible impact on the amount of intracellular eDNA that was captured, indicating that a variety of feasible pore sizes are available for targeting intracellular eDNA.
Ladin et al. (2021) [50]	Forest ecosystem	*Mycoplasma* sp., *Spirosoma* sp., *Roseomonas* sp., *Lactococcus* sp. *Spiroplasma* sp., *Methylobacterium* sp., *Massilia* sp., *Pantoea* sp., *Sphingomonas* sp.	Microbial biodiversity	This innovative technique has the potential to be used to quantify both prokaryotic and eukaryotic lifeforms by evaluating the variety of microbes in forest ecosystems
Mena et al. (2021) [113]	Tropical forests	Mammal diversity	Metabarcoding	This work is one of the first to demonstrate the enormous potential of eDNA metabarcoding for evaluating Amazonian mammal ecosystems..
Leempoel et al. (2020) [28]	Terrestrial	Mammal diversity	Diversity assessment	Ecosystem surveys could benefit from eDNA-based monitoring; however, enriching mitochondrial reference datasets is necessary first.
Rota et al. (2020) [2]	Soil	Alpine biodiversity	Metabarcoding	This research gave a description of the soil fauna of alpine habitats, produced a description of the community composition for each habitat, and revealed the relationship between the study area’s topographic features, flora, and soil characteristics
Thomsen and Sigsgaard (2019) [22]	Wildflowers	Arthropod communities	eDNA metagenomics	Genomic markers like 16S rRNA and COI can be utilized to obtain data related to arthropods from different ecological groups.
Seeber et al. (2019) [25]	Terrestrial	African mammal	Hybridization capture of eDNA	Hybridization capture enrichment of environmental DNA can be an effective technique for monitoring terrestrial mammal species.
Ficetola et al. (2019) [26]	Terrestrial	Amphibians and reptiles	Species distribution	Environmental DNA can be a valuable method to investigate terrestrial organisms, assess the relative abundance of species and distinguish amphibians and reptiles
Valentin et al. (2018) [24]	Crop surfaces	*Halyomorpha halys*	Invasive exotic insect infestations	The knowledge of environmental DNA for the surveillance of exotic species in terrestrial ecosystems can provide high sensitivity and detection.
Chang et al. (2018) [43]	Terrestrial	Migratory species	Pollinators and migratory species	The concept of environmental DNA can aid in comprehending the pollen from the migratory pollinators and the distances and geographic assortment of migratory species.
Banchi et el. (2018) [60]	Air borne and terrestrial	Fungal diversity	Species monitoring	The study revealed that diversity analysis using environmental DNA showed ten times more inclusive taxa detection than microscopic identification.
Khaliq et al. (2018) [63]	soil	*Phytophthora* diversity	Biodiversity analysis	The study revealed that environmental DNA is suitable for documenting the *Phytophthora* at the species level.
Liu et al. (2018) [70]	Terrestrial	Archeal diversity	Biodiversity monitoring	Environmental DNA can be utilized to estimate community composition based on the *mcrA* gene studies.
Galan et al. (2018) [84]	Terrestrial	bats	Diet variability	The study used the metabarcoding approach to detect the variability of the bats’ diet to cognize the bats’ biology and conservation strategies.
Gellie et al. (2017) [85]	Soil	Bacterial diversity	Species composition changes	Environmental DNA based on amplicon sequencing offers consistent ecological monitoring and cost-effective detection
Parducci et al. (2017) [64]	Soil	Palaeofloras reconstruction	Sediment profiling	Environmental DNA can be employed as a contrivance for identifying indigenous vegetation.
Bitok et al. (2017) [65]	Soil	Biosynthetic gene clusters (BGC)	BGC identification	Soil environmental DNA screening provides identification of gene clones embedding BGCs
Wakelin et al. (2016) [68]	Soil	Soil environmental genomics	High-density functional gene microarray analysis	The study revealed that environmental DNA defines the alterations in soil functional ecology and nitrogen cycling metalloenzymes.
Katz et al. (2016) [95]	Soil	Microbial diversity	Secondary metabolite screening	Environmental DNA, in combination with metagenomics, provides an alternative for natural product discovery.
Drummond et al. (2015) [101]	Soil	Alpha, beta and gamma diversity	Biodiversity assessment	Diversity estimations are affected by the number of sequence reads. Soil beta diversity exhibited the strongest response regarding elevation variation of environmental DNA markers.
Hunter et al. (2015) [97]	Terrestrial	Python diversity	Biomonitoring	The study revealed that species-specific environmental DNA assays could be used to detect python diversity.
Gibson et al. (2014) [110]	Bulk sample	Tropical arthropods	Assessment of macro and microbiomes in a bulk sample	Next-generation sequencing (NGS) can detect species in a bulk sample of terrestrial arthropods
Ramirez et al. (2014) [93]	Soil cores	Bacterial and archeal diversity	Biodiversity and biographic patterns	The study revealed that environmental DNA could be utilized to detect unscripted below-ground diversity, much of which has never been explored and explained in public databases.
Calvignac-Spencer et al. (2013) [54]	Carrion-fly derived DNA	Mammalian diversity	Biodiversity assessment	The investigation revealed that Carrion flies represent an unexploited resource of mammal DNA.
Bienert et al. (2012) [79]	Soil	Earthworms	Species identification	The study illustrates the potential of environmental DNA as a contrivance to assess the soil-dwelling diversity of animal taxa.
Anderson et al. (2012) [32]	Soil	Camels	Vertebrate diversity	The deeper portions of soil strata preserve DNA that can be an excellent indicator of the above-ground composition of the vertebrate community.

## 5. Challenges and Drawbacks

Different research networks at a dynamic scale through environmental DNA investigations drive the thought of ecologists, stakeholders and end users. However, resolving a few issues in data elucidation would be necessary to apply eDNA technologies successfully. 

The main issue with eDNA testing is the frequency of false positives (the target microorganisms are absent, but their DNA is retrieved) and false negatives (the organisms are present but cannot be recovered). One of the main issues with research seeking to discover invasive, uncommon, or perhaps endangered species is false negatives [115]. Many variables influence the chances of detection. Even while each species’ concentration of eDNA in the environment is crucial for identifying it, other variables come into play. The ability to isolate eDNA relies on the sampling effort, extraction effectiveness, analysis-impeding factors (such as PCR inhibitors), and the sensitivity of the analytical techniques [116]. False positives, however, raise concerns since they may reflect design contamination at a predetermined point in the process. In addition, issues arise from poor primer selection or design, inadequately specialized probes, or possibly ambiguities that arise throughout the sequencing process [117]. The introduction of negative controls at every stage of the analytical chain helps correct these false positives that result from technique flaws. By locating the source of contamination, this kind of strategy will be able to check the reference sequence and correct protocol flaws and sampling techniques (sample collection and filtering) [118].

The application of the environmental DNA technique is hindered by the uncertainty of the restoration monitoring method to provide abundance measures. To assess the resilience of ecosystem function, information on the abundance of individuals can be an important aspect [119]. Several reasons cause abundance biases, such as the amplification and assay selection in metabarcoding can skew sequence abundances, which can be solved with sequencing without PCR. However, the concentration of microbial DNA tends to drown out other DNA without PCR amplification to target those barcodes [120]. Another issue is the amount of eDNA deposited may be affected by the biomass of the organisms [121]. Mathematical modeling has been proposed to correct bias in metagenomics datasets, but it has been validated only for bacteria [122]. The concept of coarse proxy can be employed in restoration monitoring; however, any evaluation of population abundance will likely be taxa-dependent and may require data calibration. 

Prior to data collection, optimization of bioinformatics begins with the construction of primers [123]. In silico explanations can assist with primer design, functionality, and style and identify markers that provide an adequate resolution [124]. An ideal gene area for metabarcoding combines ease of recovery with many species in the target assemblage. To recover them from other taxa, marker qualities should maintain primer binding locations or even combine degenerate bases [125]. Primers often aim to amplify division of around 150 bp since eDNA is frequently degraded, which adds further complexity to data analysis. Contrarily, traditional DNA barcoding or metabarcoding amplicons are typically >500 bp. This helps distinguish between closely related species and often reveals intraspecies genetic changes missed by short amplicons. The important assumptions associated with using eDNA must be thoroughly and rigorously checked, particularly regarding sampling, stability, and transit [59]. Refined tactics and strengthened promotions for knowledge and comprehension will reduce pervasive skepticism, especially in the administration of the eDNA. Additionally, data from eDNA research conducted using traditional methods and produced by diverse laboratories in varied settings must be correlated [126]. The main need should be to achieve harmony between using conventions designed to handle the assessment, particularly in situations that are being explored, and developing a predictable feature of validation and standardization.

## 6. Knowledge Gaps and Future Perspectives

Environmental monitoring using eDNA is getting prodigious attention even though recently, the origin, state, transport, and fate of eDNA and the influence of these factors on species identification, quantification, data scrutiny, and result elucidation have been under the radar of researchers. Though the possibilities of eDNA methodologies are progressively clarifying, still right now, little to no genetic analysis is being conducted in management programs even though diverse experiments and trials based on the eDNA technique are being conducted across the globe. Environmental DNA and conventional survey techniques as alternative methods cannot be justified for biodiversity monitoring and management since both approaches generate dissimilar data. Investigators must reflect upon both methods’ pros and cons while addressing the queries under investigation. In that case, the eDNA approach can complement the traditional survey methods. Molecular approaches need to be made functional concurrently with in-effect procedures in crucial environmental studies, which would be expected to be completed through the improvement and testing of such approaches. Furthermore, an all-inclusive eDNA monitoring program may be conceivable from corner to corner over an assortment of environments and taxa in the near future. This will provide a global outline for ecosystem modeling and functioning for apprising biodiversity management and conservation resolutions.

In the recent years, several studies have demonstrated that eDNA in the air can be employed to detect vertebrates [98] and plants [127]. Air borne DNA (AIRDNA) can be used to track the removal of toxic plants from the rangelands, [127] indicating the potential of this method in ecological restoration monitoring. However, as with any novel method, this also needs validation before its application. The technique of eDNA will surely evolve with time, but it should not completely replace the traditional biodiversity monitoring methods rather, it must supplement it.

Similarly, researchers can employ bioinformatics tools to generate biodiversity information to improve biodiversity assessments. In contemporary times, supervised machine learning can be used to predict the biotic indices values. Such exertions have outperformed the analyses based on morpho-taxonomic assignments [128]. Furthermore, old traditional monitoring methods can benefit from innovations in bioinformatics as DNA sequences can be reanalyzed with improved reference datasets. It has been demonstrated recently by an investigation where old fish biodiversity datasets were reanalyzed. The results revealed the detection of two additional species and re-assigned a previously incorporated assigned identification for another species [129]. This study emphasizes the need for storage, curation and public availability of monitoring data and the risks of false negatives that may influence management decisions [130].

Environmental DNA with metabarcoding can support more expensive biodiversity strategies to solve these constraints. DNA metabarcoding allows examining long-term changes and local extinctions across time, especially for modeling the consequences of climate change on populations. Since eDNA metabarcoding is a novel concept, there is a need to wait for methodologies to mature before applying them to credible indices and guaranteeing backward and forward compatibility. 

The following recommendations should be adopted while dealing with environmental DNA techniques to achieve better and more reproducible results.

- Protocols should be standardized so they can be implemented globally in diverse locations of a particular habitat type.
- Development of portable instruments (qPCR, Biomeme, DNA sequencers) for rapid filed analysis to avoid the errors that may occur during sample preservation and handling.
- The data generated during environmental DNA should be properly mined to avoid reiteration. Furthermore, the mined data can be analyzed by specialists to countercheck the outcomes.

The utility of eDNA will inevitably be greatly enhanced by combining these novel advancements with time-tested field techniques normally used to assess population structure, abundance, biomass, or individual condition.

## 7. Conclusions

In biological research, the potential of environmental DNA seems complete; however, this technique demands scientific alliance and synchronization. The practical use of this innovative technique is less recurring despite having substantial potential. This study demonstrated the findings of the different studies in terrestrial ecosystems that have been carried out from 2012–2022. From the analyzed data, it can be inferred that the stepping stones to method acceptance, akin to forensic DNA analysis, might be decades in the making. We advocate that rather wait until the method is fully developed. This will surely benefit ecosystem monitoring and ecological restoration surveillance. Across different ecosystems stability of eDNA and the existence of partly disintegrated DNA in an environment needs further clarification. The present review literature indicated that the application of eDNA as a monitoring tool is increasing at a tremendous rate. However, handling and modeling ambiguities related to sampling, DNA extraction, sequencing and bioinformatics must be accurately evaluated for further investigation and standardization. Despite certain limitations, eDNA combines numerous arenas, from ecosystem restoration to human health formulating. It is remarkably an innovative monitoring tool for impending molecular investigations.

## Data Availability

All the data is included in the manuscript file.
